# Supplementary material for: Network evolution of diffusion in enterprise digitalization and intellectualization transformation: A technology—organization—environment framework perspective
Source: PLoS One. 2023 Dec 20;18(12):e0295327. doi: 10.1371/journal.pone.0295327 (PMC10732435; doi:10.1371/journal.pone.0295327)
Supplement: Attachment — Submitted filename: Response to Reviewers.docx [file pone.0295327.s002.docx]

We sincerely thank the editor and all reviewers for their valuable feedback that we have used to improve the quality of our manuscript. At the same time, we revised the article according to the format requirements and template of your journal, and added corresponding attachments and public data links for data, codes and pictures as required. The reviewer comments are laid out below in italicized font and specific concerns have been numbered. Our response is given in normal font and changes/additions to the manuscript are given in revision mode.

Reviewer #1

*Q*1: *The topic of this manuscript is very interesting, but many key factors are not taken into account in the paper. I do not agree with the research framework of Figure1 in the manuscript. Why is the technical aspect employee cost and business level? Why not consider digital technology versus existing technology? Why on the organizational side is R&D investment and technology premium?*

**Answer:** We feel great thanks for your professional review work on our article.

First, we were really sorry for our careless mistakes. In Figure 1, the technical and organizational factors are placed upside down. In that research framework of fig. 1, the correct writing should be that the technical aspect includes R&D investment and technology premium, and the organizational aspect includes staff cost and business level. The correct picture has been updated in line 193 of the manuscript.

According to your nice suggestions, we sorted out some research related to the influencing factors of digital transformation. We classify these influencing factors according to the TOE framework. The classification results are shown in Table 1. (The table has been added to the text of the manuscript)

As can be seen from Table 1, the technical aspects mainly include digital technology, AI technology and R&D. In our manuscript, with reference to Ancillo and Gavrila (2023) and Zapata et al. (2021), the first factor in technology selection is R&D investment. The second factor is the technology premium. Digital technology and artificial intelligence technology, these two words are widely used. Traditional technology mainly uses computers, networks and other hard equipment software to innovate, and its application scope has certain limitations. Digital technology is the general name of artificial intelligence, big data, block chain and other scientific technologies. Compared with traditional technologies, digital technology is more intelligent. The leading EDI transformation provides a demonstration for the enterprises in the DI transformation stage, and the enterprises in the DI transformation stage will learn from and learn from new digital technologies and business models to improve their production and operation efficiency. This process is summarized as technology premium. Technology premium, the word describes the technical advantages of digital technology itself and the influence of digital technology on traditional technology.

The Organization aspects mainly include financial resources, business level and human capital. In our manuscript, we choose staff cost as one of the organizational factors. Because staff cost can not only reflect human capital, but also reflect the characteristics of financial resources. Another factor, referring to the research of Benlian and Haffke (2016), Hansen et al (2011), Singh and Hess (2017), Jackson and Dunn-Jensen (2021), chooses business level.

**Table 1. Factors Influencing the Transformation of DI Technology at Different Levels**

| Category | | Factor | Description |  |
| --- | --- | --- | --- | --- |
| EDI | Technology  ***T*** | digital technology | Digital technology can help enterprises achieve digital management, enabling faster, more accurate, and automated production and business processes, promoting enterprise innovation, reducing costs, and improving profit margins. | Alexopoulos et al. (2022) [26]  Trung, et al. (2021) [27]  Appio et al. (2021) [28]  Tsiavos and Kitsios (2022) [29]  Al-Ruithe et al. (2018) [30] |
|  |  | AI  technology | AI technology can flexibly meet consumer needs, further expand service channels, reduce energy resource consumption in the supply chain, minimize production costs in various links, promote continuous improvement of production efficiency and profit margin, reduce the occurrence of human errors in the production process, and improve product quality. | Yeh et al. (2015) [31]  Turel et al. (2019) [32]  Weill et al, (2019) [33]  Verhoef et al. (2021) [34] |
|  |  | R&D | R&D resources refer to the human, material, and financial resources necessary for innovation activities. | Ancillo and Gavrila (2023) [35]  Zapata et al. (2021) [36] |
|  | Organization  ***O*** | financial resources | The cost of implementing and supporting the project, including procurement, delivery, hardware and software installation, staff and management training costs. | Omrani et al. (2022) [37]  Ana Kutnjak. (2021) [38]  Jones et al. (2021) [39] |
|  |  | business level | Used to measure a company's business level and development stage, which can include enterprise size, skills of management and leadership. | Benlian and Haffke (2016) [40]  Hansen et al (2011) [41]  Singh and Hess (2017) [42]  Jackson and Dunn-Jensen (2021) [43] |
|  |  | Human capital | Managers who are proficient in the basic properties of digital technology, Developing skills for existing workers and future digital workforce. | Nadkarni and pr. (2020) [44]  Holmstro and Nyle (2015) [45]  Al-Alawi et al. (2023) [46]  Hess et al (2016) [47]  Colbert et al. (2016) [48] |
|  | Environment  ***E*** | Sectorial relevance | The relationship between industry characteristics and competition, collaboration, and imitation between enterprises. | Martínez-Caro, Cegarra-Navarro and Alfonso-Ruiz (2020) [49]  Kane et al. (2015) [50] |
|  |  | government support | Introduce policies, mainly including subsidies and tax exemptions. | Wang et al. (2023) [51]  Manda and Backhouse (2018) [52]  Zhang et al. (2021) [53] |
|  |  | consumer preference | Consumer preference is a personalized preference that reflects the degree of consumer preference for different products and services, and is an important factor affecting market demand. Mainly determined by the impact of local social environment, customs, fashion changes, etc. on the entire consumer group or a specific group at that time. | Dehnert, M et al. (2022) [54] |

Reviewer #2

*This paper mainly investigates how to promote the process of EDI by complex network game theory and TOE framework. This is a hot topic problem nowadays. However, it’s a pity that the literature contribution of this version manuscript is relatively marginal. Some detailed advice is listed as follows.*

*Q*1*: You analyze the three levels of TOE technology, organization, and environment, and derive the payoff matrix providing a theoretical basis for the game model. However, the relationship between them seems very ambiguous.*

*Q*2*: In line 175, what’s the meaning of other income (such as production and operation efficiency improvement)?*

*Q*3*: EDI is an accumulation process in reality. The complex network game in this paper now can’t reflect the feature. Meanwhile, Hypothesis 2 seems not correspond to the fact of reality. As such, some analysis based on real situations should be given.*

*Q*4*: Some notations haven’t been explained clearly. Such as the equation in line 263. Hence, you’d better give a notation table to make them. Meanwhile, some terms are not explained clearly, e.g., ‘premium effect’.*

*Q*5*: Typos in Table 1. It is ‘Not Adopt’ instead of ‘Adopt’.*

We feel great thanks for your professional review work on our article.

According to your nice suggestions, we further clarify the contribution of this article. Firstly, it focuses on the micro subjects of Digitalization and Intellectualization Enterprises (DIE), deriving the conditional logic of EDI transformation based on the TOE framework of technological innovation diffusion. This research is a useful attempt to apply the toe framework to the transformation of EDI, which broadens the application boundary of the toe framework. Secondly, the study addresses the limitations of the Fermi update rule in different game situations of the complex network of EDI. By combining the savage criterion to design a renewal strategy, the study aims to improve the enterprise returns based on risk reduction. This research provides decision-making basis for enterprises to improve product pricing. Finally, the study introduces an inverted U-shaped subsidy function and a dynamic subsidy to the traditional evolutionary game model to compare the advantages of different government subsidy strategies. This approach provides a new reference for the government to promote DI reform in China. We added this part to lines 64~74.

**Answer 1**: Thank you very much for your question. We realize that in the manuscript, we focus on the single aspect of technology, organization and environment, but the relationship among them is vague.

Technology is the foundation of EDI. Enterprises that implement EDI form technological advantages by increasing R&D investment, and provide consumers with alternative and innovative product choices. The spillover of digital technology has had a demonstration effect on enterprises that implement traditional technology.

Organization is the main body of EDI. Staff resources and financial resources are both important factors in an organization, and staff cost takes both into account. The integration of digital technology into existing organizations requires employees who are proficient in DI technology, and the participation of labor force will bring about the improvement of management level.

The relationship between the three can be summarized as such a passage. Policies in the environment will guide the technological and organizational progress of enterprises. The competitive relationship in the environment will force enterprises that implement traditional technologies to improve their technical level and improve their organizational structure. The cooperative relationship in the environment can stimulate the positive effect of technology spillover and accelerate the progress of the organization. Technological progress is guided by policies in environmental factors and stimulates the industry environment to form a learning effect. The progress of the organization has created a peer effect for the industry environment.

We added this part to lines 182~189. And we integrate the relationship between the three into the research framework of Fig 1, which has been further improved.

**Fig 1.** TOE framework for the transformation and upgrading conditions of EDI

**Answer** **2**: In line 175, the specific meaning of other income in the manuscript refers to all economic income except the income from main business. These benefits are formed through daily activities such as selling goods, providing labor income and transferring the right to use assets. These benefits have the characteristics of low frequency, small amount of each business and low proportion of income. In addition, we accurately define the product income mentioned in the manuscript as the main business income (mainly through products) that enterprises can obtain by adopting traditional digital means. It has been supplemented in the description of important parameters in Table 2.

**Answer 3**: EDI is an accumulation process in reality. The income of complex network proposed in this paper is the income of all enterprises in the whole network. In the process of network game, it gradually realizes stable growth, which reflects that EDI achieves stable income in the process of continuous accumulation. Take the dynamic subsidy as an example. As shown in Figure 8a, under the dynamic subsidy, the proportion of EDI is 30%, and after the dynamic subsidy adjustment, the proportion of EDI has accumulated to 70%. At the same time, as shown in figure 8b, corporate income is also growing.

(**a**) Market diffusion results	(**b**) Average corporate earnings

**Fig 8.** Simulation results under different subsidy schemes

In Hypothesis 2, we use the specific case of new energy vehicles to further elaborate the hypothetical content. According to the theory of consumer preference, consumer behavior is determined by their preferences. When facing the choice between traditional and DI products in the market, the profits generated by different enterprise strategies can vary depending on consumers' preferences. For instance, in the early days of the launch of new energy vehicles, due to immature technology, most consumers still preferred traditional products, and traditional vehicles remained the main source of sales profits for enterprises. However, with the continuous development of DI technologies, such as intelligent driving, planning, key element perception, and energy saving, automobile consumers' preferences have shifted towards digital intelligence. These consumers are willing to pay higher prices for products with more DI characteristics. This shift has resulted in a significant increase in the proportion of sales profits from new energy vehicles in enterprises, which has further incentivized the automobile industry to prefer the production of DI products. We added this part to lines 240~255.

**Answer 4**: Important parameter Table 2 has been added to the manuscript. Compared with traditional technologies, digital technology is more intelligent. The leading EDI transformation provides a demonstration for the enterprises in the DI transformation stage, and the enterprises in the DI transformation stage will learn from and learn from new digital technologies and business models to improve their production and operation efficiency. This process is summarized as technology premium. For the premium effect, *α*_1_ is the premium factor of the product income of the DI transformation and upgrading enterprise (referring to the fact that the production of DI products can improve the corresponding product income in the process of DI transformation). *α*_2_ is the premium factor for other benefits of enterprises in DI transformation and upgrading (it means that other benefits can be improved by adopting DI technology and management means in the process of DI transformation). *α*_3_ is the premium factor for the labor cost of enterprises in the process of DI transformation and upgrading (it means that adopting DI technology and management means can reduce the corresponding labor cost in the process of DI transformation).

**Answer 5**: Thanks for your careful checks. We are sorry for our carelessness. Based on your comments, we modified the word ‘Not Adopt’ in Table 1 and checked the whole manuscript.
