# Supplementary material for: Network evolution of diffusion in enterprise digitalization and intellectualization transformation: A technology—organization—environment framework perspective
Source: PLoS One. 2023 Dec 20;18(12):e0295327. doi: 10.1371/journal.pone.0295327 (PMC10732435; doi:10.1371/journal.pone.0295327)
Supplement: Attachment — Submitted filename: Response to Reviewers.docx [file pone.0295327.s003.docx]

We sincerely thank the editor and all reviewers for their valuable feedback that we have used to improve the quality of our manuscript. At the same time, we revised the article according to the format requirements and template of your journal, and added corresponding attachments and public data links for data, codes and pictures as required. The reviewer comments are laid out below in italicized font and specific concerns have been numbered. In addition, relevant literature for which the reviewers provided references was cited in the suitable place. Our response is given in normal font and changes/additions to the manuscript are given in revision mode. At the end of the reply letter, the article needs to add financial support, the specific content is given in the reply, and the relevant content is also added in the cover letter.

*Reviewer #1:*

*The reviewer believes that the topic “Diffusion and Evolution of Enterprise Digitalization and Intelligentization (EDI) Transformation and Upgrading: A TOE Framework-based Analysis” is worthy of investigation. However, the following needs to be addressed. There are minor and major issues that should be corrected. I believe the paper could be further strengthened by added information about.*
***Q1:*** *What is the purpose of the paper? What are the research implications?*

**Answer:** We thank the reviewers for your comments, according to which the enterprise digitalization and intelligence (EDI) studied in the article is an important aspect of China's modernization process. “*Given that uncertainty in government and business decisions hinders EDI transformation, research on the evolutionary process of EDI diffusion and the strategies adopted by stakeholders in market environments is necessary. Therefore, … The findings of the study can provide valuable insights into factors that drive EDI transformation*”. The paragraph has been added to the introduction.

***Q2:*** *Please reorganize the manuscript at the journal request. Please change the reference format.*

**Answer:** Thanks to the reviewer's comments, the article has been reformatted to follow the journal format and the appendix images, data, and code files have been updated.

***Q3:*** *The language of this manuscript is very bad and needs help from native speakers.*

**Answer:** We thank the reviewers for your comments, based on which we sought the help of native speakers and revised the language of the article, as shown in the marked section of the revised paper with proof of touch-ups.

***Q4:*** *The title of the manuscript should fully demonstrate the content of this study and the relevant subjects.*

**Answer:** We thank the reviewers for your comments, and based on the review comments, the title has been revised to take into account the content and topic of the study: *Network evolution of diffusion in enterprise digitalization and intellectualization transformation: A technology–organization–environment framework perspective*.

***Q5:*** *Abstracts should include the purpose and findings of the study. Please simplify the presentation of the study process.*

**Answer:** Thanks to the reviewer's comments, according to the reviewer's comments, we revised and added the purpose and significance of the study to the abstract of the article. At the same time, we have simplified the description of the research process by revising the abstract to read “*Enterprise digitalization and intellectualization (EDI) is a crucial aspect of China’s modernization process. However, uncertainty in market and business decisions hinders the EDI diffusion process in China … They also indicate recommendations for government policies to enhance diffusion efficiency and reasonable pricing for enterprises to promote returns*”. For specific changes, please see the markup in the abstract section of the revised manuscript.

***Q6:*** *According to data released....................... This a very vague statement. These sentences do not provide any information on how the concept could be conceptualized?*

**Answer：**Thanks to the reviewer's comments, according to the reviewer's comments, the data at this place in order to lead to the importance of the subsequent concept of digital intelligence, so the following modification has been added after the paragraph as a supplement “*Data captured by digital technology needs to be further explored for its value through the application of digital intelligence technology, thus, there is an urgent need to develop concepts other than digitization.*” For specific changes, please see the paragraph markers in the revised manuscript.

***Q7:*** *Building on the preceding analysis.................... This section should explain the study's context and research objective. Furthermore, the research gap needs to be narrowed after analyzing the previous studies. The research method is not adequately explained in the first section.*

**Answer：**Thanks to the reviewer's comments, according to the reviewer's comments, we added the related research on digital intelligence in the introduction part, and further added the research on the gaming methods related to the EDI transformation and its shortcomings in that part. Meanwhile, in the first part, we add a description of the relevant research methods and propose some methodological gaps in the research. On this basis, we briefly explain the background and purpose of the study in this paragraph. Specific changes are marked in the revised manuscript.

***Q8:*** *Here author must build research gap following the previous studies. -The manuscript does not answer the following concerns: Why is it timeliness to explore such a study? What makes this study different from the previously published studies? Are there any similarly findings in line with the previously published studies? Are the findings different from prior academic studies that were conducted elsewhere, if any? What are the new technologies, some recent issue highlights the importance. See: An adoption-implementation framework of digital green knowledge to improve the performance of digital green innovation practices for industry 5.0.* [*https://doi.org/10.1016/j.jclepro.2022.132608*](https://doi.org/10.1016/j.jclepro.2022.132608)

**Answer:** Thanks to the reviewer's comments, according to the reviewer's comments and with reference to related studies, the relevant content in the article has been enriched based on the following considerations: the article is considered to address is to further propose the concept of digitization, and at the same time to study the impacts of different corporate decisions in the market during the process of digital and intelligent transformation of enterprises. The study has similarities with existing studies, i.e., both consider changes in enterprise transformation under digital or intelligent technologies, but the difference is that the existing studies only unilaterally consider digital or smart transformation, with fewer references to the new concept and technology of DI. Meanwhile, for the application of the method, most of the articles adopt the method of two-party or multi-party evolutionary game, without considering the results of the game after the formation of complex networks by different enterprises. The main parts that need to be revised have been mentioned in the introduction, and of course, the relevant parts of other parts of the article have been revised, such as the abstract and the conclusion, and the specific changes are marked in the revised manuscript.

***Q9:*** *There is no flow in the text. It partly depends on the lack of proofreading but also on the fact that many statements and claims are made without being followed up by a clear and logical discussion. It is especially problematic in the Introduction that brings up a number of findings from different areas without linking them together.*
**Answer:** Thanks to the reviewer's comments, according to the reviewer's comments, the article organized the logic of the introduction part, according to the logic of "research background - digitalization or intelligence research - DI research - EDI transformation research - research deficiencies - existing research methods - research deficiencies - research objectives - research significance" modified the introduction and added part of the literature enriched the relevant content. At the same time, each part is organized according to the depth of the literature research content, and the specific changes are marked in the introduction of the revised manuscript.

***Q10:*** *More importantly, the choice of the questionnaire questions should be explained in light of the theory and the prior literature on the topic. The arguments are simply relationships and causes very close to the replication of many studies dealing with the same thing. For example, what is connection of upgrading path of manufacturing enterprises from the value perspective. See the following: The governance mechanism of the building material industry (BMI) in transformation to green BMI: The perspective of green building. https://doi.org/10.1016/j.scitotenv.2019.04.317*
**Answer:** Thanks to the reviewer's comments, according to the reviewer's comments and with reference to related studies, we have made the following considerations: “*First, at the technical level, technology R&D investment will enhance the degree of specialization of enterprises for the application of DI technology. Simultaneously, the research and development of new technologies will also enable the enterprise to enhance the corresponding knowledge base, and accumulate knowledge of DI transformation. The technology premium effect has a positive impact on the economic consequences of product returns, production, and operational efficiency, and the economic foundation determines the superstructure, which can increase investment in the research and development of digital intelligence technology and the introduction of relevant talent with the support of a sufficient economy. Second, at the organizational level, human resources play a crucial role in promoting EDI transformation. Human resources are an important cost for enterprises, which can not only promote the transformation of knowledge results generated by R&D investment, but may also reduce labor costs via the introduction of DI technology. Third, at the environmental level, enterprises are not only in a competitive relationship with similar firms but also affected by heterogeneous consumers in the marketplace and government policies. Consumers’ consumption concepts advance the important driving force of EDI transformation, which can source operating funds as well as enhance the enterprise’s reputation and promote the sustainable development of the enterprise. Simultaneously, only by adhering to government policies can we better seize opportunities in the international environment, realize the social value of enterprises, and meet the needs of national economic development*”. The paragraph has been added to the second subsection of Part 2 with relevant references, as indicated in the revised manuscript marker.

***Q11:*** *Methodology: Model. I suggest authors here build your main heading on Research and data methodology. Clearly explain the model building process, and what previous studies have used similar models (model testing approach).
See the following: A three-player game model for promoting the diffusion of green technology in manufacturing enterprises from the perspective of supply and demand.https://doi.org/10.3390/math8091585
A stochastic differential game of low carbon technology sharing in collaborative innovation system of superior enterprises and inferior enterprises under uncertain environment, https://doi.org/10.1515/math-2018-0056
Information fusion for future COVID-19 prevention: continuous mechanism of big data intelligent innovation for the emergency management of a public epidemic outbreak.*
**Answer:** Thanks to the reviewer's comments, according to the reviewer's comments and with reference to related studies, we have revised the title of the fourth and fifth parts of the article in conjunction with the content of the study, and added the process of modeling and the related study of applying the model at the beginning of the fourth part. The specific modifications are shown in the markup of the fourth and fifth parts of the revised manuscript.

***Q12:*** *Please consider this structure for manuscript final part. -Discussion-Conclusion-Managerial Implication-Practical/Social Implications*
**Answer：**Thanks to the reviewer's comments, we have reorganized the article according to the structure suggested by the reviewers, specific textual changes are reflected in the markup of the revised manuscript.

***Q13:*** *Please make sure your conclusions' section underscores the scientific value-added of your paper, and/or the applicability of your findings/results. Highlight the novelty of your study. In addition to summarizing the actions taken and results, please strengthen the explanation of their significance. It is recommended to use quantitative reasoning comparing with appropriate benchmarks, especially those stemming from previous work.*

**Answer：**Thanks to the reviewer's comments, according to the reviewer's comments, we further emphasize the scientific value and novelty at the beginning of the conclusion. Meanwhile, in the new dicussion section, we take government subsidies and corporate pricing as a point of discussion to explore their further value in the light of previous studies, and the specific textual changes can be found in the markups in Part 5 and 6 of the revised manuscript.

The article needs additional financial support as follows: Specific grant numbers: Project of Humanities and Social Sciences Foundation of the Ministry of Education (Specific grant numbers: 18XJAZH004); Initials of authors who received each award: H H; Full names of commercial companies that funded the study or authors: Ministry of Education of China; Initials of authors who received salary or other funding from commercial companies: W Z; URLs to sponsors’ websites: https://www.sinoss.net/. The funders had no role in study design, data collection and analysis, decision to publish, or preparation of the manuscript.
